# Potentials of Plasma NGAL and MIC-1 as Biomarker(s) in the Diagnosis of Lethal Pancreatic Cancer

**DOI:** 10.1371/journal.pone.0055171

**Published:** 2013-02-01

**Authors:** Sukhwinder Kaur, Subhankar Chakraborty, Michael J. Baine, Kavita Mallya, Lynette M. Smith, Aaron Sasson, Randall Brand, Sushovan Guha, Maneesh Jain, Uwe Wittel, Shailender K. Singh, Surinder K. Batra

**Affiliations:** 1 Department of Biochemistry and Molecular Biology, University of Nebraska Medical Center, Omaha, Nebraska, United States of America; 2 Eppley Institute for Research in Cancer and Allied Diseases, University of Nebraska Medical Center, Omaha, Nebraska, United States of America; 3 Department of Biostatistics, University of Nebraska Medical Center, Omaha, Nebraska, United States of America; 4 Department of Surgery, University of Nebraska Medical Center, Omaha, Nebraska, United States of America; 5 Department of Internal Medicine, University of Nebraska Medical Center, Omaha, Nebraska, United States of America; 6 Division of Gastroenterology, Hepatology and Nutrition, University of Pittsburgh School of Medicine, Pittsburgh, Pennsylvania, United States of America; 7 Departments of Gastroenterology, Hepatology, and Nutrition, UT MD Anderson Cancer Center, Houston, Texas, United States of America; 8 Department of General and Visceral Surgery, Universitätsklinik Freiburg, Freiburg, Germany; Technische Universität München, Germany

## Abstract

Pancreatic cancer (PC) is lethal malignancy with very high mortality rate. Absence of sensitive and specific marker(s) is one of the major factors for poor prognosis of PC patients. In pilot studies using small set of patients, secreted acute phase proteins neutrophil gelatinase associated lipocalin (NGAL) and TGF-β family member macrophage inhibitory cytokine-1 (MIC-1) are proposed as most potential biomarkers specifically elevated in the blood of PC patients. However, their performance as diagnostic markers for PC, particularly in pre-treatment patients, remains unknown. In order to evaluate the diagnostic efficacy of NGAL and MIC-1, their levels were measured in plasma samples from patients with pre-treatment PC patients (n = 91) and compared it with those in healthy control (HC) individuals (n = 24) and patients with chronic pancreatitis (CP, n = 23). The diagnostic performance of these two proteins was further compared with that of CA19-9, a tumor marker commonly used to follow PC progression. The levels of all three biomarkers were significantly higher in PC compared to HCs. The mean (± standard deviation, SD) plasma NGAL, CA19-9 and MIC-1 levels in PC patients was 111.1 ng/mL (2.2), 219.2 U/mL (7.8) and 4.5 ng/mL (4.1), respectively. In comparing resectable PC to healthy patients, all three biomarkers were found to have comparable sensitivities (between 64%-81%) but CA19-9 and NGAL had a higher specificity (92% and 88%, respectively). For distinguishing resectable PC from CP patients, CA19-9 and MIC-1 were most specific (74% and 78% respectively). CA19-9 at an optimal cut-off of 54.1 U/ml is highly specific in differentiating resectable (stage 1/2) pancreatic cancer patients from controls in comparison to its clinical cut-off (37.1 U/ml). Notably, the addition of MIC-1 to CA19-9 significantly improved the ability to distinguish resectable PC cases from CP (p = 0.029). Overall, MIC-1 in combination with CA19-9 improved the diagnostic accuracy of differentiating PC from CP and HCs.

## Introduction

Despite decades of research, the prognosis for pancreatic cancer (PC) remains dismal, with an overall five-year survival rate of only about 5% [1]. A significant contributor to the poor prognosis of PC is the fact that the cancer often remains undiscovered until an advanced stage. Available techniques for the diagnosis of PC present several difficulties chiefly their invasive nature, need for specialized training, observer bias and the high cost to the healthcare system. Further, it has been demonstrated through experiments in animal models that molecular changes precede the appearance of changes in pancreatic architecture (detected by imaging techniques) [2]. Hence, there has been a growing emphasis on the identification of molecular markers that can identify PC at an early and potentially resectable stage. Body fluids, such as blood, urine, bile and pancreatic juice represent a promising source of potential biomarkers. Currently, the only biomarker that is approved for use in PC is CA19-9 which is recommended to follow the progression of PC, but not for diagnostic use. We have previously reported that neutrophil gelatinase associated lipocalin (NGAL), a 24 kDa glycoprotein, is differentially upregulated during the progression of PC. Further, in a small set of samples we showed that plasma NGAL levels were significantly elevated in PC patients compared to healthy controls [3]. Recently, El-Mesallamy et al. observed significant increase in NGAL levels in PC patients with pre-existing diabetes (142 ng/ml) in comparison to diabetic patients (66.7 ng/ml) and non-diabetic healthy controls (37.8 ng/ml). With sensitivity and specificity of 75% and 87% in differentiating PC from non-PC cases NGAL came up as potential adipokine [4]. Macrophage inhibitory cytokine (MIC-1), a distant member of the transforming growth factor β (TGF-β) family of cytokines, was originally identified as a gene expressed in the context of macrophage activation [5]. In a previous study, it was shown to be differentially expressed in PC tissues and elevated in the serum of PC patients compared to both healthy controls and those with benign pancreatic neoplasms [6]. Further, Ozkan et al., observed significantly elevated expression of MIC-1 in PC cases in comparison to other pancreatobilary diseases and healthy controls. It was found to have similar sensitivity as that to CA19.9 (81%) in differentiating PC from other benign diseases [7]. Serum MIC-1 was found to outperform CA19-9 in CA19-9, in differentiating patients with resectable pancreatic cancer from controls [8], [9].

In the present study, we sought to extend the findings from our pilot study to investigate the diagnostic utility of plasma NGAL in PC [3]. Given the participation of both MIC-1 and NGAL in inflammation and the close relationship between inflammation, chiefly of the chronic nature, and PC [3], we added MIC-1 to the panel of potential biomarkers. Plasma CA19-9 was used as a reference to compare the diagnostic performance of both NGAL and MIC-1. Our results suggest that plasma levels of both NGAL and MIC-1 were significantly elevated in patients with PC. In the present study, MIC-1 was found to be highly specific in distinguishing patients with surgically resectable PC (i.e. early stage 1/2) from CP cases. Improved diagnostic efficacy of CA19-9 was observed in differentiating stage 1/2 PC patients from HCs at an optimal cut-off >54.1 U/ml (74% sensitive and 92% specific) in comparison to its clinical cut-off (37.1 U/ml) (71% sensitive and 67% specific). Finally, multivariate analysis revealed that a combination of plasma MIC-1 and CA19-9 is significantly superior to CA19-9 alone in differentiating resectable PC from CP (AUC = 0.85 vs. 0.74, p = 0.029).

## Materials and Methods

### Study Design

This retrospective dual center study for plasma markers in PC was approved by the Institutional Review Boards (IRB) of the University of Nebraska Medical Center (UNMC) (IRB number 209-00) and the University of Pittsburgh Medical Center (IRB number PRO07030072). Written informed consent was obtained from all patients and controls before enrollment into the study. Inclusion criteria was any adult patient (age ≥18 years) with histologically proven PC that that was admitted to the University of Pittsburgh during the period from 2002 to 2009. Chronic pancreatitis (CP) was defined based on CT scan findings of calcifications, abnormal pancreatogram or secretin stimulation test. For this study, 91 PC, 23 CP patients and 24 healthy controls were enrolled. Baseline demographic information for all groups is detailed in **Table 1**.

**Table 1:** Demographics and clinicopathologic characteristics of patients included in the study.

Variable	HC	PC	CP	p-value
**N (%)**	24 (17.4%)	91 (66%)	23 (16.6%)	
**Mean (SD) age**	56 (6.7)	65.5 (10.6)	62.6 (11)	0.0005
**Males (%)**	4 (18%)	55 (60%)	14 (61%)	0.0013
**Race**				
(i) White	20 (91%)	55 (92%)	21 (91%)	0.39
(ii) Black	0 (0%)	4 (7%)	1 (4%)	
(iii) Asian	2 (9%)	1 (2%)	1 (4%)	
(iv) Missing	2	31	-	
**Smoker**				
(i) Ever	2 (25%)	56 (62%)	-	0.063
(ii) Never	6 (75%)	35 (38%)	-	
(iii) Missing	16			
**BMI**		25.6 (5.5)		
**Stage**				
1B		5 (6%)		
2A		6 (7%)		
2B		31 (38%)		
3		2 (2%)		
4		38 (46%)		
Missing		9		
**Location of tumor**				
(i) Head		64 (71%)		
(ii) Body		16 (17%)		
(iii)Tail		8 (9%)		
(iv) Uncinate process		2 (2%)		
(v) Missing		1		
**Grade of tumor**				
(i) Well differentiated		7 (11%)		
(ii) Moderately differentiated		30 (45%)		
(iii) Poorly differentiated		29 (44%)		
(iv) Missing		25		
**Family History of PC**				
Present		8 (10%)		
Missing		8		
**History of DM-II**		25 (27%)		

BMI; Body Mass Index; DM-II: Diabetes Mellitus type II; SD: Standard Deviation.HC: Healthy Controls; PC: Pancreatic Cancer; CP: Chronic Pancreatitis.

For PC patients, a sample was classified as “treatment naive” if the sample was drawn prior to any cancer-directed surgical or chemotherapeutic intervention. For diagnostic analyses, only treatment naïve samples were used. PC staging was based on one of four criteria: 1) pathological staging post-surgery 2) MRI/CT/ultrasound staging if this was the only staging available, 3) endoscopic staging if the patient never underwent surgery or 4) biopsy of metastatic disease if no previous staging was available. PC grade, location of the tumor, stage, smoking status, history of type 2 diabetes and family history of PC were based upon review of hospital records.

### Determination of Plasma NGAL and MIC-1 by Sandwich ELISA

NGAL and MIC-1 levels in plasma were measured quantitatively by sandwich ELISA according to the manufacturer’s instructions using the DuoSet ELISA kit (R&D Systems) for human NGAL and MIC-1 respectively. The plasma samples were stored at −70°C immediately following receipt and aliquoted to avoid repeated freeze thaw cycles. Standard curves were produced from NGAL and MIC-1 standards provided with the kit and serially (log_2_) diluted from 4 ng/ml to 15.6 pg/ml. All measurements were done in triplicates and samples with readings greater than that of the highest standard were diluted appropriately and the assay was repeated. ELISA plates were read at 450 nm with an absorbance correction at 540 nm. Data collected was analyzed using the SOFTMAX PRO software (Molecular Devices Corp., Sunnyvale, CA).

### CA19-9 Radioimmunoassay Assay

CA 19-9 antigen concentration was determined by a solid phase radioimmunoassay (Centocor, Malvern, PA, USA), using the manufacturers recommendation. All samples were analyzed in duplicate and the quantities of CA 19-9 were expressed in arbitrary units (U/ml) where one unit activity corresponds to approximately 0.8 ng of purified antigenic protein for CA 19-9 in a solid phase radioimmunoassay [10].

### Statistical Analysis

Data was analyzed using SAS statistical software version 9.2 (SAS Institute Inc, Cary, NC). Age, body mass index (BMI), plasma levels of CA19-9, NGAL and MIC-1 were analyzed as continuous variables, while gender, race, tumor grade, cancer stage, and surgical status were considered to be categorical. Patient characteristics were compared between PC, CP and control patients using ANOVA model for continuous variables and chi-square tests or Fisher’s exact test wherever applicable. Biomarkers were analyzed on the natural log scale due to the skewed nature of the data. Biomarkers were compared between groups using t-tests and ANOVA models. If significant differences were found in the overall p-values from the ANOVA models then pairwise comparisons were made adjusting for multiple comparisons with Tukey’s method.

Logistic regression was used to create ROC (receiver operating characteristic) curves, which are used to describe the performance of biomarkers as diagnostic tests that are measured on a continuous scale. ROC curves are presented using the biomarkers to predict the diagnosis of pre-treated (i.e. no surgery or chemotherapy) PC from controls. The area under the curve (AUC) was used to evaluate the usefulness of a biomarker as a diagnostic test. Odds ratio (OR) were calculated as a measure of the strength of association between a biomarker and the disease condition under study. For all univariate analyses, PC was considered the disease state, with CP or healthy controls considered the control group. Multiple logistic regression was used to evaluate the performance of the biomarkers in combination as a predictors of pre-treated PC compared to controls. P-values ≤0.05 were considered statistically significant.

## Results

### Elevated Levels of NGAL, MIC-1, and CA19-9 in Pancreatic Cancer Patients

Plasma samples from a total of 138 subjects were analyzed comprised of 91 PC (66%), 23 CP (17%) and 24 healthy control (HC, 17%) subjects. PC and CP patients were significantly older than HCs (p = 0.0005) and had a higher percentage of males (60% and 61% vs. 18%, p = 0.001). A majority of study subjects were Caucasians (92%, 91% and 91% of PC, CP and HC respectively). Sixty-two percent of PC and 25% of HC patients were ever smokers. Nearly half of PC patients had resectable tumors (51%). A majority of the tumors (71%) were located in the pancreatic head and were moderate to poorly differentiated (89%). Ten percent of PC patients had a family history of PC while 27% had a history of type 2 diabetes (**Table 1**). Assessment of the correlation of serum bilirubin with CA19.9 level in both CP and PC cases revealed no correlation between CA19.9 and bilirubin levels for PC (r = 0.179 p = 0.080) and CP cases (r = 0.459 p = 0.042). For the present study, due to the skewed distribution of biomarker levels, each biomarker measurement was log transformed (into its natural logarithm, to the base *e* = 2.7183) prior to comparison of mean levels between the three groups of patients. For the purposes of presentation, data has been reverse-log transformed to allow the inclusion of units. The intra and inter-assay percent coefficient of variation (% CV) for NGAL and MIC-1 were 4.1%, 14.3%, 5.9% and 16.1% respectively. Due to the presence of high and low standards built into the commercial kit, these coefficients were not determined for CA19-9.

The mean plasma concentration (after log transformation) of NGAL, MIC-1 and CA19-9 were all significantly higher in PC patients (111.1 ng/mL, 4.5 ng/mL, and 219.2 U/mL) than in the healthy controls (67.4 ng/mL (p = 0.01), 1.5 ng/mL (p = 0.003), and 31.5 U/mL (p = 0.001)). Additionally, serum concentration of MIC-1 and CA19-9, but not NGAL, were found to be higher in the PC patient group than in CP patients (1.6 ng/mL (p = 0.003), 31.8 U/mL (p<0.001), and 111.1 ng/mL (P>0.05) respectively) (**Table 2**).

**Table 2:** Comparison of biomarker levels between patient groups.

	Healthy Control (n = 24)		Pancreatic Cancer# (n = 91)		Chronic pancreatitis (n = 23)		
	Mean	SD	Mean	SD	Mean	SD	p-value
**CA19-9 (U/mL)**	31.5	1.48	219.2	7.77	31.8	2.80	<0.0001
**NGAL (ng/mL)**	67.4	1.52	111.1	2.23	111.1	2.14	0.013
**MIC-1 (ng/mL)**	1.5	4.85	4.5	4.10	1.6	1.80	<0.0001

SD: Standard deviation. #PC patient samples were limited to treatment naïve samples only for this analysis.

NGAL levels were significantly higher in patients aged 60 years or more (p = 0.045). MIC-1 levels were significantly lower in ever smokers compared to never smokers (p = 0.021). CA19-9 levels on the other hand were significantly elevated in female PC patients and in those with unresectable disease (Stage 3/4, p = 0.045 and 0.0047 respectively) (data not shown).

### Diagnostic Accuracy of NGAL, CA19-9 and MIC-1

**Figure 1:**
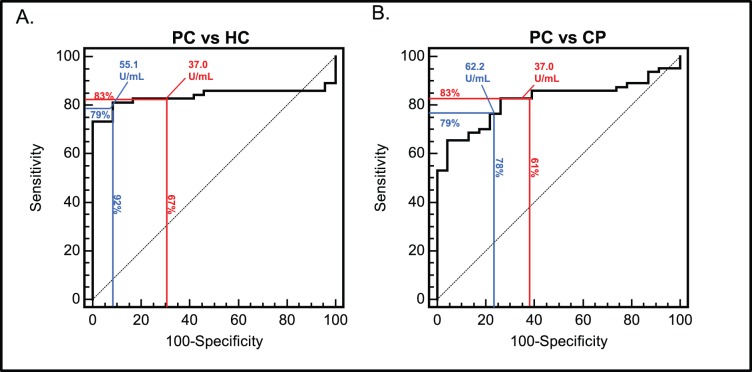
Reciever Operating Chacteristic curves comparing CA19-9 at its clinical cut-off of 37 U/ml and optimal cut-off of 55.1 U/ml in treatment naïve group. ROC curve analyses for assessing the ability of CA19-9 to differentiate PC form HC (panel A) and PC from CP (panel B) at its clinical cut off (37 U/ml) (red line), and optimal cut-off (55.1 U/ml) (blue line). At a cutoff of 37 U/mL, CA19-9 differentiated treatment naïve PC patients from healthy controls with sensitivity, specificity of 83% and 67%, while sensitivity and specificity of 79% and 92% was observed at 55.1 U/ml. In case of PC vs CP patients, sensitivity and specificity of 61% and 83% were observed at clinical cut-off while at optimal cut-off 62.2 U/ml sensitivity increased to 79% with specificity of 78%.

We next sought to investigate the sensitivity and specificity of the three biomarkers for diagnosing PC. PC patients were divided either based on disease stage or treatment status. As post-treatment samples are not diagnostically relevant, only treatment naïve samples were included in these analyses. In order to check diagnostic efficacy of CA19-9, MIC-1 and NGAL, these markers were evaluated at predefined cut-off of ≥37 U/ml, ≥1.07 ng/ml, ≥106 ng/ml as observed in earlier studies [3], [6]. During this validation, NGAL was found to be 92% sensitive while MIC-1 was most specific (94%) in distinguishing early stage 1/2 patients from healthy controls (**Table 3**). However, overall performance of all the markers was quite poor. Further, we evaluated their diagnostic efficacy at optimal cut-off. For CA19-9, apart from the commonly employed cut-off value of ≥37 U/ml, we also used optimal cut-off (55.1 U/ml) as determined by ROC curve analysis. In comparison of both PC to HC and PC to CP patients, use of an higher cut-off of CA19-9 resulted in higher specificity with similar sensitivity in distinguishing PC from either CP or HCs (**Figure 1**) (**Table 4**). For all the further analysis, we used CA19-9 at its optimal cut-off 55.1 U/ml. Notably, CA19-9 at its optimal cut-off was 79% sensitive and 92% specific in distinguishing treatment naive PC patients from HCs. MIC-1 was the most sensitive (81%) and CA19-9 the most specific marker (92%) distinguishing resectable PC patients (stage 1/2) from HCs. For distinguishing resectable PC patients from CP patients, MIC-1 was the most specific (78%) marker and NGAL was the most specific marker (100%) in distinguishing the stage 3 and 4 PC group from CP cases.

**Table 3:** Diagnostic potential≠ of NGAL, MIC-1 and CA19-9 at pre-defined cut-off.

Groups	Pre-definedcut-off	Sensitivity	Specificity
**PC vs. HC**			
CA19-9	≥37 U/ml	83%	67%
MIC-1	≥1.07 ng/ml	90%	46%
NGAL	≥106 ng/ml	42%	92%
**PC vs. CP**			
CA19-9	≥37 U/ml	83%	61%
MIC-1	≥1.07 ng/ml	90%	30%
NGAL	≥106 ng/ml	42%	52%
**Stage 1/2 PC vs. HC**			
CA19-9	≥37 U/ml	71%	67%
MIC-1	≥1.07 ng/ml	94%	46%
NGAL	≥106 ng/ml	46%	92%
**Stage 3/4 PC vs. HC**			
CA19-9	≥37 U/ml	88%	67%
MIC-1	≥1.07 ng/ml	90%	46%
NGAL	≥106 ng/ml	44%	92%
**Stage 1/2 PC vs. CP**			
CA19-9	≥37 U/ml	71%	61%
MIC-1	≥1.07 ng/ml	94%	30%
NGAL	≥106 ng/ml	46%	52%
**Stage 3/4 PC vs. CP**			
CA19-9	≥37 U/ml	88%	61%
MIC-1	≥1.07 ng/ml	90%	30%
NGAL	≥106 ng/ml	44%	52%

≠ PC patient samples were limited to treatment naïve samples only for this analysis.

**Table 4:** Determining optimum cut-off of CA19-9, NGAL and MIC-1 for diagnosis of pancreatic cancer^≠^.

Groups	Optimum cut-off	Sensitivity	Specificity	OR from cut-point	95% CI	p-value
**PC vs. HC**						
CA19-9	≥37 U/ml	83%	67%	8.4	2.8–25.8	0.0002
ln (CA19-9)	>55 U/ml	79%	92%	40.7	8.2–203	<0.0001
ln (MIC-1)	>2.3 ng/ml	62%	63%	2.7	0.97–7.4	0.056
ln (NGAL)	>83 ng/ml	67%	57%	2.7	0.97–7.5	0.058
**PC vs. CP**						
CA19-9	≥37 U/ml	83%	61%	6.6	2.2–19.9	0.0009
ln (CA19-9)	>62.2 U/ml	79%	78%	13.3	4.0–44.8	<0.0001
ln (MIC-1)	>2.3 ng/ml	62%	78%	5.8	1.8–18.4	0.0028
ln (NGAL)	>157.6 ng/ml	34%	65%	0.97	0.3–2.8	0.95
**Stage 1/2 PC vs. HC**						
CA19-9	≥37 U/ml	71%	67%	7.3	2.4–22.6	0.0005
ln (CA19-9)	>54.1 U/ml	74%	92%	31	6.2–153.9	<0.0001
ln (MIC-1)	>2.2 ng/ml	81%	64%	6	1.9–18.2	0.0018
ln (NGAL)	>91.8 ng/ml	64%	88%	12.6	3.2–49.3	0.0003
**Stage 3/4 PC vs. HC**						
CA19-9	≥37 U/ml	88%	67%	18	4.7–68.5	<0.0001
ln (CA19-9)	>54.1 U/ml	83%	92%	51.9	9.8–273	<0.0001
ln (MIC-1)	>1.6 ng/ml	78%	58%	4.8	1.6–14.5	0.005
ln (NGAL)	>86.5 ng/ml	58%	79%	5.1	1.6–16.5	0.006
**Stage 1/2 PC vs. CP**						
CA19-9	≥37 U/ml	71%	61%	5.7	1.9–17.4	0.0022
ln (CA19-9)	>49.4 U/ml	76%	74%	9.1	2.8–29.2	0.0002
ln (MIC-1)	>2.3 ng/ml	76%	78%	11.5	3.4–39	<0.0001
ln (NGAL)	>70.8 ng/ml	76%	30%	1.4	0.4–4.36	0.56
**Stage 3/4 PC vs. CP**						
CA19-9	≥37 U/ml	88%	61%	14	3.7–52.9	0.0001
ln (CA19-9)	>186 U/ml	70%	96%	51.3	6.2–425	0.0003
ln (MIC-1)	>3.5 ng/ml	55%	91%	12.8	2.6–62.2	0.0015
ln (NGAL)	>-28.5 ng/ml	5%	100%	ND	ND	ND

HC: Healthy Controls; CP: Chronic Pancreatitis; PC: Pancreatic Cancer; ln: natural log; ND: Not Determined.^≠^PC patient samples were limited to treatment naïve samples only for this analysis.

### Diagnostic Accuracy of a Combination of Two or Three Markers for PC Compared to CA19-9 Alone

**Figure 2:**
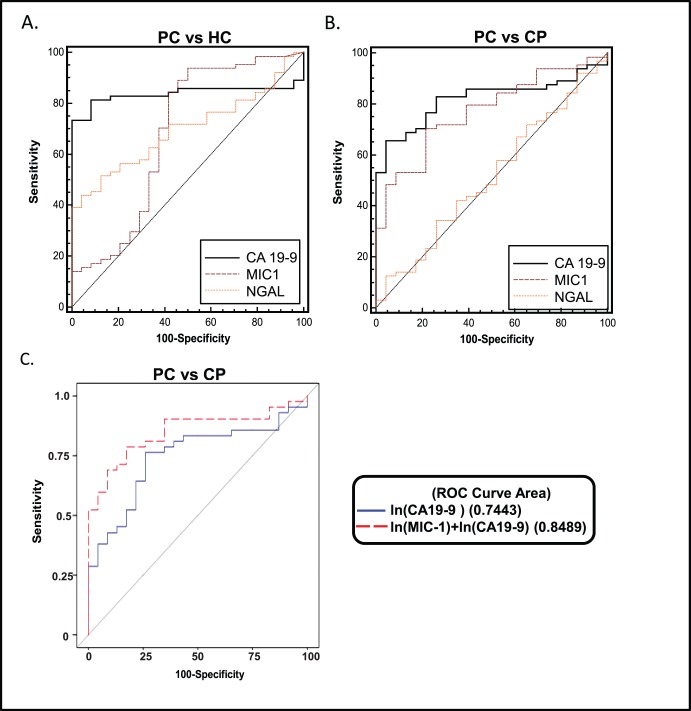
Reciever Operating Chacteristic curves comparing NGAL, MIC-1, CA19-9 for differentiating PC from HC (panel A) and CP (panel B) and combination of CA19-9 and MIC-1 in differentiating treatment naïve resectable PC from CP. The combined use of MIC-1 with CA 19-9 significantly improved the sensitivity and accuracy in differentiating resectable PC from CP (AUC = 0.85 Vs 0.74, p = 0.029, C.I. 0.76–0.94) in comparison to CA19-9 alone.

CA19-9 is currently the only FDA approved biomarker that is used to aid in the diagnosis and to follow the progress of PC patients. Having examined the diagnostic performance of individual markers, we next sought to investigate whether adding either NGAL or MIC-1 to CA19-9 (the gold standard) improved the ability to distinguish PC cases (resectable or unresectable) from CP or HCs. The combination tests were determined via multivariate analyses of individual markers, and those showing statistically significant differences between respective patient groups being used further for analysis. Addition of NGAL and MIC-1 improved the area under the curve (AUC±SE) from 0.8 (0.06) to 0.85 (0.05) in distinguishing stage1/2 PC from HCs (**Table 5**). The addition of NGAL (with or without MIC-1) had significant impact on the ability of CA19-9 to distinguish PC (either resectable or unresectable) from HCs. Notably, the addition of MIC-1 to CA19-9 significantly improved the ability to distinguish resectable PC cases from CP (AUC 0.74 with CA19-9 alone and 0.85 with the combination (C.I. 0.76–0.94) (**Figure 2**) (**Table 5**). Further, addition of NGAL improved the AUC from 0.89 (0.05) to 0.94 (0.03) in distinguishing stage 3/4 PC from HCs.

**Table 5:** Comparison of Area under the ROC curve for NGAL, MIC-1 and CA19-9 in the diagnosis of pancreatic cancer^≠^.

Groups	AUC (SE)	95% CI	p-value^a^
**Stage 1/2 PC vs. HC**			
ln CA19-9	0.8 (0.06)	0.69–0.91	
ln NGAL+ln CA19-9	0.82 (0.05)	0.72–0.93	0.4
ln NGAL+ln CA19-9+ ln MIC1	0.85 (0.05)	0.75–0.94	0.22
**Stage 3/4 PC vs. HC**			
ln CA19-9	0.89 (0.05)	0.80–0.98	
ln NGAL+ln CA19-9	0.94 (0.03)	0.87–1.00	0.11
ln NGAL+ln CA19-9+ ln MIC1	0.94 (0.03)	0.89–1.00	0.13
**Stage 1/2 PC vs. CP**			
ln CA19-9	0.74 (0.06)	0.62–0.87	
ln MIC-1+ ln CA19-9*	0.85 (0.05)	0.76–0.94	0.029
ln NGAL+ln CA19-9+ ln MIC1	0.86 (0.04)	0.77–0.95	0.027
**Stage 3/4 PC vs. CP**			
ln CA19-9	0.87 (0.04)	0.79–0.96	
ln MIC-1+ ln CA19-9*	0.93 (0.03)	0.87–0.99	0.079
ln NGAL+ln CA19-9+ ln MIC1	0.92 (0.03)	0.86–0.99	0.12

PC (pancreatic cancer), CP (chronic pancreatitis), AUC (area under the curve), SE (standard error).^ a^P-value against CA19-9 alone.*Marker inclusion in combination tests was based on statistical significance of differentiation of individual biomarkers levels by multivariate analysis). ^≠^PC patient samples were limited to treatment naïve samples only for this analysis.

## Discussion and Conclusions

The management of PC would greatly benefit from an early diagnostic biomarker. Various potential candidates have been tested but none have been consistently found superior to CA19-9. Recently, our group observed *de novo* expression of NGAL (acronym for neutrophil gelatinase associated lipocalin), a 25 kDa secreted glycoprotein, in PanIN-1s, the earliest premalignant lesions preceding PC. Further, we noted in a limited set of patient samples that serum NGAL levels were significantly elevated in the plasma of patients with CP and PC compared to healthy individuals (p = 0.035 and 0.004 respectively) [3]. Koopman et al, had reported in an earlier study that another small cytokine MIC-1 (macrophage inhibitory cytokine 1) could distinguish PC patients from HCs with a sensitivity and specificity of 94% and 90% respectively [6]. As both MIC-1 and NGAL are small, secreted glycoproteins, we hypothesized that a combination of these two markers could discriminate PC patients from HCs and CP patients. To investigate this hypothesis, we compared their diagnostic accuracy with that of CA19-9, the current gold standard marker for PC. The results of our study suggest that NGAL and MIC-1 may be at least comparable to CA19-9 in their diagnostic accuracy in specific situations. Specifically, MIC-1 has sensitivity and specificity similar to CA19-9 (at its optimal cut-off) in distinguishing treatment naïve PC patients from CP patients (**Table 4**). Further, the addition of MIC-1 (with or without NGAL) improved the ability to distinguish both resectable (stage 1 or 2, p = 0.029) and unresectable (stage 3 or 4, p = 0.079) PC from CP patients (**Table 5**).

NGAL and its murine homologue Ngal have been proposed as components of the innate immune system [11]–[13]. In our earlier studies, we observed that overexpression of NGAL in PC cells inhibit invasion and metastasis and prevents angiogenesis [14]. The observation that NGAL levels are similar in CP and PC patients (**Table 2**) suggests that NGAL may be released as a part of the chronic inflammatory response that accompanies both diseases.

MIC-1 (also called as GDF-15 or NAG-1) is a member of the TGF-β family that was first identified as a protein secreted from macrophages in response to immune activation [15]. MIC-1 is also aberrantly expressed by several malignancies (including PC) and has emerged as target of p53 mediated transcription (role of MIC-1 in cancer reviewed in [15]). Differential expression of MIC-1 was observed in SAGE (serial analysis of gene expression) libraries from six pancreatic cancer cell lines in comparison to non-neoplastic tissues [16]. Koopman and colleagues had reported earlier that MIC-1 was significantly better than CA19-9 in discriminating PC from HCs (AUC being 0.99 and 0.78, p = 0.003) but not from CP (AUC being 0.81 and 0.74 respectively, p = 0.63) [6]. They observed that the mean MIC-1 level in healthy controls, CP and PC was 0.76 ng/ml, 2.36 ng/ml and 5.4 ng/ml respectively. Further studies emphasized the diagnostic efficacy of MIC-1 equivalent to CA19-9 [6], [7]. In our study, the mean plasma MIC-1 levels in these patient groups were 1.5 ng/ml, 1.6 ng/ml and 4.5 ng/ml (**Table 2**). In our sample set, at an optimal cut-off of >2.3 ng/ml, plasma MIC-1 was 62% sensitive and 63% specific in discriminating PC from HCs. At this cut-off, MIC-1 was 78% specific and 62% sensitive in differentiating PC from CP patients. Interestingly, the combined use of MIC-1 with CA 19-9 significantly improved the sensitivity and accuracy in differentiating resectable PC (Stage 1/2) patients from CP patients (AUC of 0.85, p = 0.029) in comparison to CA19-9 alone (AUC of 0.74), providing a promising approach for PC diagnosis at an early stage. The significance of MIC-1 as a biomarker for PC will need to be investigated in larger patient cohorts.

CA19-9 is a well-known molecular marker in PC. Biochemically, it is the sialylated Lewis antigen present on several glycoproteins. Overall, it has a reported sensitivity and specificity of between 70%–80% and 70%–90% respectively [17], [18]. However, its major drawback is that it can also be positive in several benign conditions [1], [19]. In our study, we observed that CA19-9 at the commonly employed cut-off of >37 U/ml was 83% sensitive and 67% specific in distinguishing PC patients from HCs and 83% sensitive with 61% specificity in differentiating PC patients from CP. Interestingly, the optimal cut-offs for CA19-9 as a diagnostic marker (55 U/ml, 79% sensitive and 92% specific in distinguishing PC patients from HCs and 62.2 U/mL, 79% sensitive and 78% specific in differentiating PC patients from CP) were higher than the common clinically employed cut-off of 37 U/ml. These higher cut-offs, though yielding similar sensitivities, increased the specificity of CA19-9 (**Table 4**). This observation suggests that no one single cut-off for CA19-9 (or for that matter any other biomarker) is applicable to all situations. Additionally Morris-stiff et al., observed that elevated levels of CA19-9 correlated directly with the degree of biliary obstruction (*r* = 0.911, *P*<0.001) but for malignant diseases CA19-9 levels were elevated independent of bilirubin (*r* = 0.117288, *P* = 0.603) [20]. Similarly, we assessed the correlation of serum bilirubin with CA19.9 level in both CP and PC cases. However, no correlation was observed between CA19.9 and bilirubin levels for PC (r = 0.179 p = 0.080) and CP cases (r = 0.459 p = 0.042). Larger studies in the future will aim to assess the impact of each of these cut-offs in distinguishing specific groups.

A limitation of the present study is the lack of information on the prognostic significance of NGAL and MIC-1 in PC. Elevated NGAL levels have been reported in earlier studies to correlate with reduced survival in ovarian [21] breast [22] and gastric cancer [23] patients while MIC-1 levels did not show any correlation with survival in oesophageal cancer [24] but, when elevated in the cerebrospinal fluid, was associated with a shorter survival in patients with glioblastomas [25]. Additionally, the study involved 51% of the PC patients with resectable (Stage 1/2) tumor (early stage tumor), a scenario quite different from clinics. Thus diagnostic efficacy of these markers might vary according to the population set. Future studies will seek to answer this question to better delineate the clinical applicability of these biomarkers in the management of PC.

The strength of our study is the rigorous design and techniques used. Specifically, Koopman and colleagues had employed ELISA to measure both CA19-9 and MIC-1. However, clinically, CA19-9 levels are commonly measured by radioimmunoassay (RIA). Initially, we tried employing ELISA to measure CA19-9 to try and keep our methodology as similar to the earlier study as possible. However, we noted that the measurements using ELISA lacked reproducibility (data not shown). Hence, we then re-analyzed all samples for CA19-9 levels by RIA. Thus, the study was not only an investigational study but also served to validate previous studies. Based on our observations, we agree with employing the RIA technique for reproducible measurement of CA19-9.

In summary, we have investigated whether quantitative measurement of NGAL, MIC-1 and CA19-9 could be useful in the diagnosis of PC. We observed that while the level of all three biomarkers was significantly elevated in PC in comparison to HCs, only CA19-9 and MIC-1 were significantly elevated in CP patients compared to HCs. Log transformed value of NGAL was more specific than CA19-9 in distinguishing stage 3/4 PC patients from CP cases while that of MIC-1 was more sensitive (stage 1/2 PC from HCs) or specific (stage 1/2 vs CP) than CA19-9 in a sub-group specific manner. CA19-9 performed better in distinguishing PC form CP patients or HCs at a higher cut-off value than the commonly employed cut-off of 37 U/ml. A combination of MIC-1 and CA19-9 was better than the latter alone in distinguishing resectable PC from CP patients while addition of NGAL improved the ability of CA19-9 to distinguish stage 3/4 PC cases from HCs.
